# Detection of Silver Nanoparticles inside Marine Diatom *Thalassiosira pseudonana* by Electron Microscopy and Focused Ion Beam

**DOI:** 10.1371/journal.pone.0096078

**Published:** 2014-05-05

**Authors:** César Pascual García, Alina D. Burchardt, Raquel N. Carvalho, Douglas Gilliland, Diana C. António, François Rossi, Teresa Lettieri

**Affiliations:** 1 European Commission—Joint Research Centre, Institute for Health and Consumer Protection, Ispra (VA), Italy; 2 European Commission—Joint Research Centre, Institute for Environment and Sustainability, Ispra (VA), Italy; 3 FU-Berlin, Fachbereich Biologie, Chemie, Pharmazie, Berlin, Germany; 4 Departamento de Biologia and CESAM, Universidade de Aveiro, Aveiro, Portugal; King Abdullah University of Science and Technology, Saudi Arabia

## Abstract

In the following article an electron/ion microscopy study will be presented which investigates the uptake of silver nanoparticles (AgNPs) by the marine diatom *Thalassiosira pseudonana*, a primary producer aquatic species. This organism has a characteristic silica exoskeleton that may represent a barrier for the uptake of some chemical pollutants, including nanoparticles (NPs), but that presents a technical challenge when attempting to use electron-microscopy (EM) methods to study NP uptake. Here we present a convenient method to detect the NPs interacting with the diatom cell. It is based on a fixation procedure involving critical point drying which, without prior slicing of the cell, allows its inspection using transmission electron microscopy. Employing a combination of electron and ion microscopy techniques to selectively cut the cell where the NPs were detected, we are able to demonstrate and visualize for the first time the presence of AgNPs inside the cell membrane.

## Introduction

Diatoms play a major role in the earth's carbon cycle fixing about 40% of the total carbon in oceans and serve as the base of the marine food chain [1]. They are ubiquitously distributed in all aquatic ecosystems and have been used for many years as ecological indicators [2]–[4]. More recently, diatoms have also been studied as non-model organisms to investigate the mechanism of toxicity of chemical pollutants at a molecular level [5]–[7]. Given the increasing relevance of engineered nanomaterials worldwide and their uncontrolled release into the environment there is a rapidly growing concern about the potential toxicological impact on many aquatic organisms including small autotrophs such as diatoms. Among the various classes of nanomaterials present in commercial products silver nanoparticles (AgNPs) represent one of the most commonly used due to their highly efficient antibacterial properties. Common examples of their use include biocidal additives, silver impregnated antibacterial materials, and disinfectants. Nanoparticle incorporation has been reported in various cell types such as algae [8], nematode [9], fish [10], [11] and human mesenchymal stem cells [12] although the uptake mechanisms and affected intracellular pathways are still under investigation. Previous research has suggested that the toxicity of AgNPs to microorganisms is due to the release of Ag ions into the media [13], [14]. In a recent study of time-dependent cellular growth, we reported how exposure to both AgNPs and silver nitrate (AgNO_3_) could inhibit the growth of diatoms and cyanobacteria. The data suggested that the toxicity for the biota was the result of a combination of effects from both the AgNPs and the released silver ions [15].

The unique morphology of diatoms with their silica outer shell represents a particular challenge for the study of AgNPs internalization. The marine diatom *Thalassiosira pseudonana* used in our studies is characterized by a cylindrical shell consisting of two valves joined by girdle bands-features which have been widely characterized by techniques like scanning electron microscopy (SEM) or atomic force microscopy (AFM) [16], [17]. The silica outer shell is assembled in an intricate three-dimensional pattern of nanopores leaving a considerable surface open for the interaction of the cell with its environment, including the transport of nutrients or even environmental pollutants. The diffusion of particles through the diatom outer shell has been shown to depend not only on the overall surface of the cell but also on the size of the pores and the tortuosity of the path [18]. Thus, the intricate three-dimensional structure likely represents a natural filter for the flow of larger molecules and nanomaterials such as NPs.

A number of metal NPs have unique plasmon-resonant optical scattering properties that can be used to localize them inside microorganisms using optical microscopy [19]. The identification and localization of intracellular gold NPs has been possible with advanced methods such as Raman or hyperspectral confocal microscopy [20], [21]. The detection of the characteristic plasmonic signature of silver NPs in the diatom/AgNPs system provided indications that a similar situation may be occurring in this system [22]. Unfortunately, more precise localisation of NPs in cells is not possible by optical microscopy because of the fundamental limitations in resolution imposed by the wavelength of the incident light.

Recently, study using a combination of optical and AFM methods showed the interaction of NPs with diatom cells [22]. They demonstrated evidence that nanometer scale structural changes to cell morphology can be induced by AgNPs and inferred the internalization of AgNPs into the cell following the observation of coagulation of the internal cell material. Unfortunately, the application of AFM is restricted to the study of the outer shell and thus the presence of NPs inside could not be visualized with submicron resolution.

Transmission electron microscopy (TEM) has been employed to image the interaction of polydispersed AgNPs with the bacteria *Ochromonas danica* and *Pseudomonas putida* [8], [23]. This methodology involves the preparation of cell slices with a thickness of around 100 nm making the study of whole cell morphology difficult since consecutive sequences of microtome slices must be imaged if a complete tomography of the cell is to be achieved. SEM instrumentation, when compared to TEM, is generally simpler and more accessible and this has been widely employed to characterize the external morphology of diatoms [24], [25]. Furthermore, SEM in combination with X-ray spectroscopy can provide very good resolution of the morphology as well as the spatial distribution of the atomic elements in diatoms [26]. To target the inside of the diatom SEM can also be used in combination with ion-abrasion employing a focused ion beam (FIB) [24]. When suitably automated this method allows the 3D reconstruction of organic tissues with a good results in terms of depth versus resolution [27]. The principle problem of TEM microtome and FIB 3D reconstruction methods is that the processes of cell cutting is relatively slow and this is further compounded by the fact that the position of the NPs in a sample fixed with resins is *a priori* unknown, so finding the sections of interest occurs by trial and error or by a long systematic series of cuts.

Recently, we have presented a convenient method of using electron and ion microscopy [28], [29] to study the interactions of metal NPs with cells grown in suspension. The method, based on cell preparation by critical point drying, was able to detect NPs down to 5 nm in the cells and to distinguish between genuine uptake/internalization and mere interactions between the particles and the membrane surface. In our studies, we used this technique to investigate the internalization of the AgNPs in the marine diatom *T. pseudonana*. The original method was adapted to use diatoms as a way to adequately preserve the shape of the organism while, at the same time, ensuring the necessary transparency for the electron microscopy study in transmission mode.

In our procedure the localisation of the NPs in the diatom was based on the comparison of the signals from the transmitted and scattered electrons. In this way the NPs which were detected in transmission but not with SEM mode were below the surface. To confirm their presence inside the cytoplasm of the diatoms we applied FIB milling to visualize inside the cell. The silver content of nanoparticles was verified in our experiments using electron dispersion X-ray signal (EDX). To our knowledge this is the first time that it has been possible to show the presence of AgNPs actually inside the cytoplasm of the marine diatoms.

## Materials and Methods

### Materials

All experiments were carried out using maltose-stabilized nanoparticles in an aqueous suspension. Chemicals used for diatom culture media were purchased from Sigma-Aldrich. Silver nitrate (AgNO_3_) (99.9%), ammonium hydroxide (5N in H_2_O), D-(+)-maltose monohydrate (98%), sodium borohydride (98%) were purchased from Sigma-Aldrich and used without any further purification.

### Synthesis of Silver Nanoparticles

AgNPs were prepared via a modified Tollens process in which the complex cation [Ag(NH_3_)_2_]^+^ is reduced to form silver metal nanoparticles through chemical reduction by sugars as described previously [15]. The particle size distribution in the resulting AgNPs dispersion was analyzed by Centrifugal Liquid Sedimentation (CLS) [31] and Scanning Electron Microscopy (SEM). According to the particle number distribution, the sample was polydispersed with size distribution between 5 and 120 nm (data shown in supporting information).

### Diatom Culture

*T. pseudonana* (strain CCMP 1335) was purchased as axenic culture from the Provasoli-Guillard National Center for Culture of Marine Phytoplankton (CCMP, West Boothbay Harbour, Maine, USA). Diatom cultures were kept in artificial sea water (ASW)-f/2 medium [32], [33] at 14°C under a diurnal light cycle of 13 h light and 11 h darkness and continuous shaking at 100 rpm. Cell density and cell growth were calculated as published previously [34].

### Exposure of Diatom Cultures to AgNPs

In order to test the uptake of AgNPs, diatoms were exposed to silver nanoparticles at a concentration of 50 µM in ASW-f/2 medium. Diatom cells were cultured at an initial cell density of 0.75×10^6^ cells/mL in 20 mL batch cultures. After 24 h, the nanoparticles were added to the culture just before the light cycle started and then incubated for 48 h.

### Sample preparation for electron microscopy

The cell preparation for electron microscopy consisted in a standard, single cell chemical fixation [24], [35] followed by critical point drying. After incubation with AgNPs, 20−50×10^6^ cells were harvested and fixed by adding glutaraldehyde at a concentration of 2.5% for 30 min at 4°C. The cells were pelleted and washed with distilled water. The cell pellet was then post-fixed with 2% OsO_4_ for 20 min, centrifuged and washed again with water. The OsO_4_ was used to fix the lipidic structures in the cell [36]. All the centrifugation steps were performed at 2500×g for 5 min.

After cell fixation, a sequential solvent exchange was done using increasing concentrations of ethanol in water (25, 50, 75, 90 and 100%). After each solvent exchange, the cells were incubated for 5 min followed by centrifugation at 2500×g for 5 min. The final pellet was placed in a vial with a porous lid and CO_2_ critical point drying performed. Ethanol was exchanged by liquid CO_2_ at 5°C and 50 bar using several rinsing steps in an EMITEC critical point dryer. The pressure was increased in the closed volume, raising it to the critical point of CO_2_ by increasing the temperature. The pressure was then gradually decreased to ambient level over a period of one hour. The final product was a fine black powder containing the cells. Each of the samples was then transferred into individual grids of 300 µm mesh copper coated with graphite for support.

### Scanning electron microscopy and EDX analysis

A double beam scanning electron microscope FEI-NOVA 600i NANOLAB equipped with FIB and a gas injector (GIS) with platinum precursor was used for SEM analysis. The system was equipped with traditional secondary and in-lens detectors as well as X-ray analyser for Energy-Dispersive X-ray Spectroscopy sensitive to carbon and other higher atomic weight elements. A scanning transmission electron microscopy (STEM) detector was used for transmitted electrons.

In order to study the cell-NPs interaction, the cells were firstly scanned in the transmission mode to detect AgNPs using 30 keV acceleration voltage (HV) which was the maximum possible in our scanning microscope. Although traditional TEM instrumentation provides acceleration voltages up to 300 keV in our case we compared STEM images at 30 keV with TEM images taken with a JEOL JEM 2100 TEM microscope at 200 keV (see Figure S3). As would be expected, the contrast and resolution of TEM images were superior to that of STEM but the main information necessary to detect AgNPs was equivalent. After identifying the position of the NPs, the surface of the cell was explored using low accelerating voltages (5 keV) to avoid signals coming from deep intracellular structures. For those cells with insufficient electrical grounding (enough contact with the electrical conductive grid support), it was necessary to raise the energy to 25 keV to visualize the diatom surface, even though at this energy some signal from below the surface was observed. However, even when using an accelerating voltage of 25 keV the position of the surface bound NPs was recognizable using the SEM mode.

In those cases when NPs detected in transmission mode could not be found at the surface, a search for NPs inside the cell was initiated in that region by performing a cut using focused ion beam (FIB) milling. Before starting the cut, the region of the cell containing the NPs was protected by depositing a metallic layer on top of the cell using Pt-GIS activated with the electron gun to avoid a curtain effect [37]. The rest of the milling procedure was made following the method already described in [28]. EDX image maps were treated using the open software program Image-J [38] for noise reduction using a Gaussian Spatial filter (see supplementary information).

## Results

### Surface analysis of *Thalassiosira pseudonana* exposed to AgNPs

**Figure 1:**
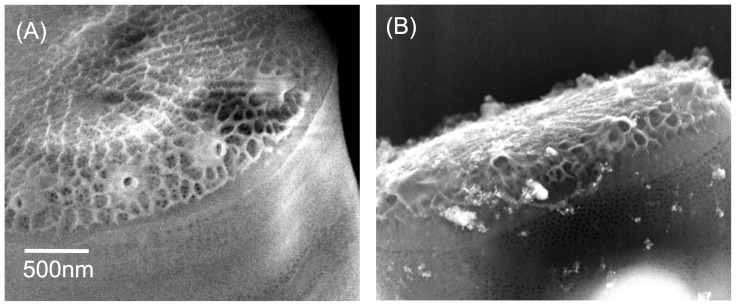
Scanning electron microscope images of the nanostructured pores in the shell of two diatoms corresponding to the control (A) and AgNPs exposed (B) samples respectively (scale bar common in both pictures).

Diatom cells exposed to AgNPs were analyzed by SEM. Figure 1A shows a representative image of the diatom external frustule belonging to the control (sample incubated without NPs) using classical SEM imaging of electrons originating mostly from the first few nanometers at the diatom surface. The untreated diatom cells showed nanopores with an average diameter of 20±3 nm (*n* = 81) while the larger pores in the valve (portulae) had an external diameter of 72±19 nm (*n* = 10). The values are consistent with those reported previously by Hildebrand et al. 2006 [16]. Figure 1B shows NPs attached to the surface of a diatom of the exposed sample. Agglomerated and dispersed AgNPs of different sizes were found on the surface of the valves and the girdle band (Figure 1B) with the latter being often associated with organic material. No noticeable morphological changes were detected in the size and shape of the diatoms when exposed to the AgNPs. Additionally, the pore sizes of the diatoms in both the untreated and exposed cells showed similar values.

**Figure 2:**
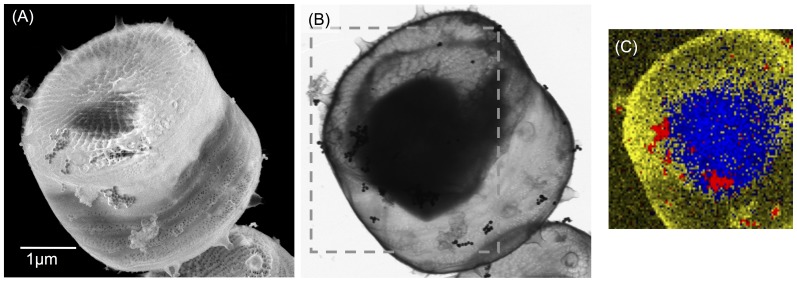
Scanning electron microscope images of a diatom exposed to AgNPs using the signal from the surface (A) and in transmission (B). (C) shows the EDX map of carbon (blue), silicon (yellow) and silver (red) of the area indicated by the dashed squares in (B) assigned to the organic part, the exoskeleton and AgNPs respectively (scale bar common to the pictures (A) and (B)).

Figure 2 shows the silver NPs detection scheme. The NPs were detected at the surface using scattered and secondary electrons in SEM mode (Figure 2A). The cell was also inspected in transmission mode with 30 keV (Figure 2B). The comparison of these two images shows where the NPs were localized. In the case of this cell, all the NPs found were at the surface. Transmission images like the one in Figure 2B allow us also to visualize the dimensions of the cytoplasm of the cell compared to the silica shell. We noticed a shrinkage of the cells of approximately 60% (estimation based on imaging shown in Figure S2), similar to other values in literature of cells treated with critical point drying [30]. The silver content of the NPs was further confirmed with EDX. Figure 2C shows the carbon (blue), silicon (yellow) and silver (red) maps, which correspond to the position of organic matter, the diatom shell and AgNPs respectively.

### Uptake of AgNPs

**Figure 3:**
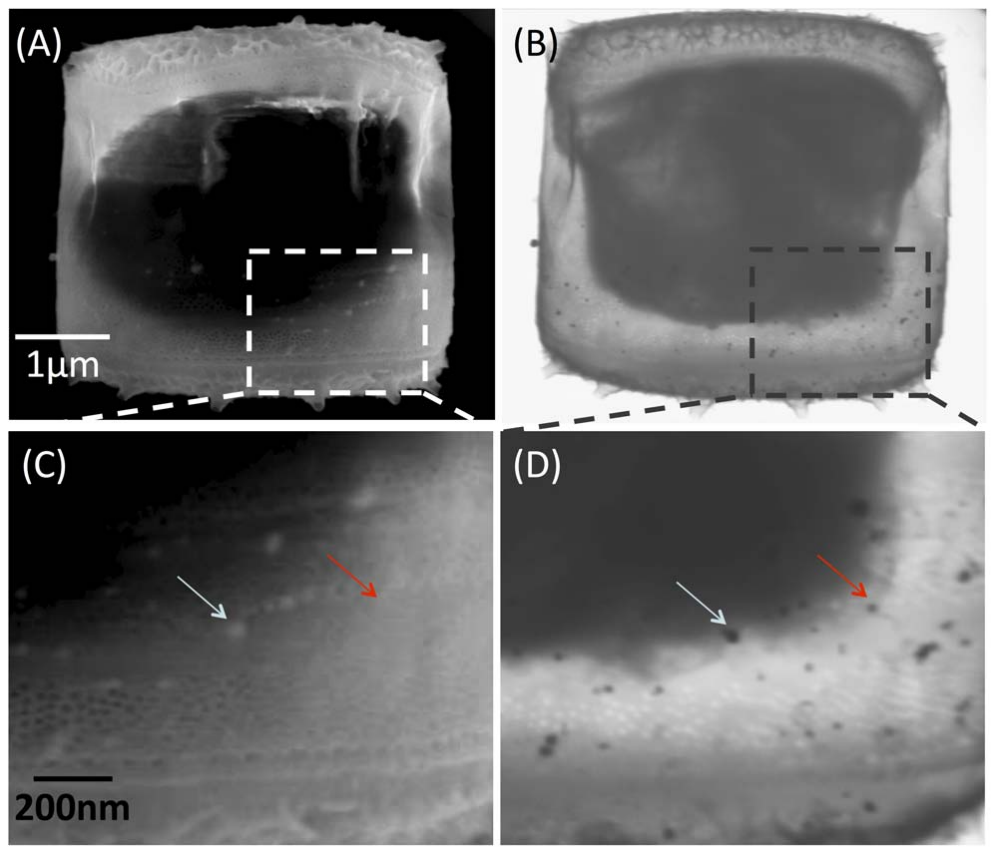
Scanning electron microscope images of a second diatom exposed to AgNPs, using the signals from the surface (A) and in transmission (B). (C) and (D) show magnifications of the same area of the cell indicated with the dashed squares, where it is possible to detect NPs in the surface (i.e. blue arrow) or potential internalized particles (i.e. red arrow) (scale bars common to pictures (A), (B) and (C), (D) respectively).

In order to study the fate of the NPs, we analyzed several diatom cells using the same approach described for the cell in Figure 2. Traditional SEM and STEM analyses of a representative diatom are shown in Figure 3 A and B, respectively. At low voltages (5 keV, Figure 3A), the signal reveals the details from the surface of the diatom. AgNPs standing outside the valve are detected in this way while the membrane and internal compartments were not visible. By contrast, using STEM at 30 keV it was possible to visualize the inner part of the diatom (Figure 3B) and screen the AgNPs in the cell that were not visible at the surface. Figures 3 C and D show an enlargement of the dashed area in Figures 3 A and B. These images were used for the localization of AgNPs. It was possible to assign the NPs to the diatom surface whenever the NPs were visible in both SEM and STEM images (e.g. see the blue arrow in Figures 3 C and D). However, some NPs were only visible in transmission mode (e.g. NPs pointed by the red arrow in Figure 3D) and were likely inside the diatom shell. These cells were selected as candidates for further analysis on NP uptake.

### Intracellular analysis of AgNPs

**Figure 4:**
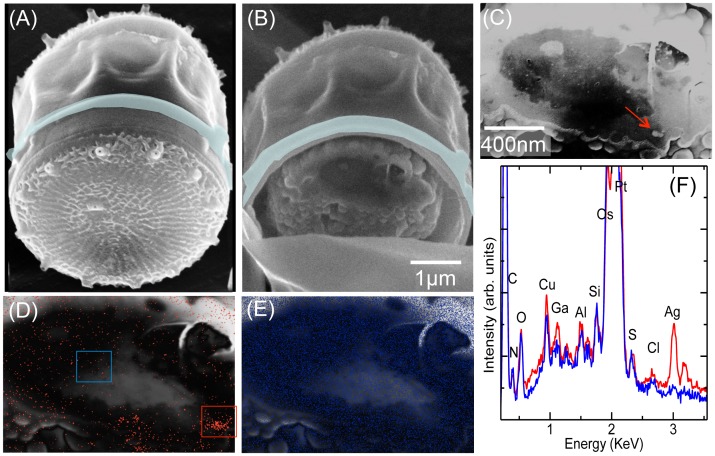
Sequence of the cut and detection of Ag content in the section from the same cell shown in **Figure 3**. (A), the cell after the deposition of the Pt protective layer. (B), the cell after the cut; (C) an enlargement of the section of the cell with an enhanced contrast. (D) and (E), the Ag and Os EDX maps respectively over the Secondary Electron signal collected by the EDX detector. (F) EDX spectra from the background (blue) and the bright spots (red) corresponding to the blue and red square area shown in the panel (D). The bright spots are also marked by the red arrow in panel (C).

To confirm the uptake and the intracellular location of silver nanoparticles, a cross-cut was performed by milling into the cell in the region where the comparison between surface and transmission images indicated the presence of NPs inside the shell of the diatom. Figure 4 illustrates a cut of the cell shown in Figure 3 done for intracellular inspection. The section was performed at the level of the cytoplasm. The platinum stripe, deposited to avoid the curtain effect during the milling process, can be seen in Figure 4A shadowed with blue. Figure 4B shows the section of the cell after the cut, while the enlargement of the cytoplasmic region is shown in Figure 4C. Some of the brighter spots localized in this picture reveal higher scattering cross sections that could indicate higher density atoms related to a silver content. Figures 4 D and E show the Ag and Os EDX maps respectively over the secondary electron signal obtained with the EDX detector. Since osmium had been used for cell fixation (see Material and Methods), the homogeneous distribution over the cytoplasm shown in Figure 4E was as expected. In contrast to this, the Ag distribution was heterogeneous (see Figure 4D), with certain areas being exceptionally dense, and matching the brighter areas in Figure 4C indicated by a red arrow. The EDX point spectra analysis in this region was compared to the background (red and blue squares in Figure 4D respectively) and plotted in Figure 4F. The elemental analysis of the cell by EDX showed signals for C, N, O, Si, Os, S and Cl from the cell as well as Cu, Al, Pt and Ga identified as contaminant signals likely coming from the supporting grid, the microscope chamber, the protection layer and deposited Ga atoms during the ion milling process, respectively. The homogeneous distribution of Os in the EDX maps confirmed the overall distribution and also indicates that no Os agglomerates were formed during the fixative process. By analyzing the SEM images of the section and the EDX data we can correlate the studied high-density clusters in Figure 4C (pointed with the red arrow) to silver aggregates.

These results could be reproduced in other cells (see Figures S5, S6). We performed different cuts in a total of 12 diatom cells. Bright spots inside the cytoplasm of the cell could be detected in seven of the cuts. These spots may be associated with Ag aggregates, although Ag EDX signal associated with them was detected only in five of the cells (a second example is shown in Figure S6). The emergence of bright spots without EDX signal could occur since the detection of X-rays is much less efficient than that of electrons, therefore there is a higher threshold for the detection of silver by EDX. In some cells we found neither Ag EDX signal nor bright spots corresponding to possible NPs, even when the comparison of the intact cell by SEM/STEM suggested that some of the NPs were located internally (e.g. in Figure S5H). The failure to detect Ag nanoparticles in the cuts may be partially explained either by the induced ion abrasion damage or by the displacement of the section due to charging effects.

## Discussion

In this study, the intracellular uptake of AgNPs by the marine diatom *Thalassiosira pseudonana* was investigated by combining SEM/STEM/FIB. By correlating the surface and transmission signals, we demonstrate that it is possible to detect NPs that have crossed the outer silica shell and then, by using an ion beam to mill into the sample, we found evidence confirming the presence of internalized metal rich clusters. The Ag content of these clusters was demonstrated using EDX analysis. The combination of dual beam microscopy with EDX is a powerful strategy to analyze a precise smooth section [24], [39]. However, with the current SEM/FIB instrumentation, the FIB abrasion requires time-consuming processes to minimize damage in the organic material of the cell. Even using the method described here which minimizes the time for milling by allows the detection of regions of interest prior to the cut, we believe that SEM/FIB methods should be considered most suitable for studies on laboratory-cultured cells rather than for cells originating in the natural environment. In the latter case the concentration of AgNPs would general be much lower making its detection difficult.

While this study has shown the presence of internalized Ag clusters, determining the mechanism by which they may reach the cell interior is a much more complex issue and requires careful consideration of many aspects of the behavior of silver nanomaterials. Metallic AgNPs may not be chemically stable under common environmental conditions and can be easily oxidized, particularly when exposed to sources of light or, alternatively, may complex with naturally occurring organic ligands such as humic or fulvic acids. Similarly, silver in the ionic state can also be reactive and can complex with organic molecules [40] such as proteins or even be reduced back to the metallic state by mild reducing agents. Reactions can occur between ionic silver and sulfides or chlorides resulting in the formation of low solubility products such as AgCl and Ag_2_S. In the case of AgCl the situation is further complicated as exposure to a high salinity environment may result in the formation of more soluble complex chlorides which can re-solubilize the Ag [15]. Many types of AgNPs are dependent on electrostatic repulsion for their colloidal stability and thus tend to aggregate in high ionic solutions such as seawater which effectively screens the charge on the particle leading to reduced repulsion and eventual coagulation due to van der Waals forces [41]. The AgNPs in our culture could be detected attached to the cell surface of *T. pseudonana*, on the valve and girdle band. While AgNPs in a monodispersed form were observed, aggregates or clusters of AgNPs were also very common. The nanoparticles found attached to the diatom cells had a broad range of sizes, including sizes smaller than the pore diameters of the outer shell. The cell wall in the centric diatom *T. pseudonana* may be permeable to small particles entering through the naturally existing pores. Nevertheless, the mobility of molecules inside a diatom pore is affected not only by the diameter of the outer opening but also by the pore geometry across the shell structure [18]. Studies performed by Yang et al. would suggest that the diatom surface topography helps the diatoms to sort and filter the particles [42]. It is thus not certain what the size exclusion limit is for the entry of nanoparticles in *T. pseudonana*.

Another mechanism that would allow the crossing of the AgNPs through the cell wall was shown by Pletikapić and coauthors using atomic force microscopy [22]. The cell exoskeleton of diatoms is covered by an organic envelope essentially composed by polysaccharides and proteins [43], [44], [45]. AgNPs have shown high reactivity with these exopolymeric substances (EPS). Indeed the production of EPS is increased with the AgNPs exposure [46], [47]. Furthermore they showed that the mechanism of entrance of AgNPs through the cell wall of the marine diatoms *C. fusiformis and C. closterium* involved localized damage without disintegration of the cell wall. In our case, AgNPs also seemed to be preferentially associated with the organic content at the surface of the *T. pseudonana* although no damage was verified in the exoskeleton, AgNPs could potentially cross the shell barrier in a similar way to that in the study by Pletikapić and coauthors.

After crossing the outer-shell in diatoms, NPs must also cross the cellular membrane to reach the cytoplasm and the intracellular compartments. In our paper we have demonstrated the presence of Ag clusters inside the cytoplasm. In a previous paper the toxic effect of AgNPs exposure to *T. pseudonana* could be explained by a combination of integral AgNPs and other silver containing species that were released in the artificial seawater [15]. The observation of intracellular nanoparticulate silver in the present study could thus explain the contribution of AgNPs to the observed toxicity by providing a localized source for the release of silver ions in close vicinity to the molecular targets. However, the SEM images and detection of silver by EDX only provide static temporal information on the presence of high density silver clusters inside the diatom cells but do not provide all the information about the mechanism of uptake or the nature of the internalized silver. In fact, our data could be explained by an uptake of small silver nanoparticles but it cannot be excluded that these-high density silver signals could arise from an uptake of free silver that subsequently precipitates and aggregates inside the cells.

In summary, we have shown a potentially attractive method for investigating the interaction of diatoms with AgNPs. We believe that its use in this study has begun to reveal more about the role of AgNPs in the toxicity of microorganisms and it shows a potential which could be exploited in further in-depth studies of their interaction with monodispersed AgNPs and ionic silver.

## Supporting Information

Figure S1 (A) SEM of the NPs used in the study. (B) Centrifuge Liquid Sedimentation analysis of the size of the AgNPs. Particles were analyzed using CLS and SEM. Particles' diameters resulted less than 120 nm. Below 30 nm CLS reaches its limit of sensitivity and we cannot exclude particles of smaller size. For this reason we considered our nanoparticles as polydispersed AgNPs.
(TIF)

Figure S2 **SEM images of one of the diatoms from the control sample using the signal from the surface (A) and in transmission (B) (common scale bar).** The control sample was prepared in the same way described in materials and methods without the incubation with AgNPs. The shrinkage of the cells was calculated from STEM images in Figures 3B and S2B using the ratio of the volume of the shell and cell-membrane. The shape used to estimate both volumes was a cylinder (Lx2π(D/2)^2^), where L and D are the measured length and diameter of the diatom corresponding to the vertical and horizontal directions respectively in both pictures.
(TIF)

Figure S3 **Transmission electron microscope (TEM) image performed with 200 keV HV.** Picture S3 shows a TEM image of a AgNP exposed diatom. The picture was made using a JEOL JEM 2100 TEM microscope at 200 keV (see Figure S3). We compared the STEM images at 30 keV with the ones at 200 keV from the TEM. The contrast and resolution of TEM images were superior to STEM, but the main information about the localization of NPs was equivalent. The higher contrast in TEM images allows a better recognition of organelles in the diatom.
(TIF)

Figure S4 **EDX Ag map before (A) and after (B) image processing using a Gaussian filter shown in the table S1.** Images were processed using open software Image-J using the convolve command.
(TIF)

Figure S5 **Electron microscope pictures of the total 12 cuts made in this study.** Bright spots in the cytoplasm region of the section were found in a total of seven cases (A) to (G), while EDX signal associated with these spots were detected in five cases (A) to (E). We performed different cuts in a total of 12 diatom cells. Bright spots inside the cytoplasm of the cell, associated with high density clusters could be detected seven of the cuts, while Ag EDX signal associated with some of these spots was detected in five of them (a second detailed example is shown in Figure S6, while for the other cuts the raw data are summarized in S7). We attribute the fact that we see more bright spots than EDX signal to the higher cross section for electron-electron scattering than electron-X-rays scattering, and therefore there is a higher threshold for the detection of silver by EDX. In some cells no high density clusters or Ag EDX signal were visible inside the cell after the cut, even though the comparison of the intact cell by SEM/STEM suggested some of the NPs were not located at the surface. This could be partially explained by the introduction of damage by the ion abrasion, as evidenced by the detection of a curtain effect (the transport of material from the top of the cell). In these cases we did not detect neither silver nor the existence of high density spots.
(TIF)

Figure S6 **Sequence of the cut and detection of silver content into AgNPs exposed diatom.** (A) and (B) are scanning electron microscope images of the diatom incubated with AgNPs using the signal from the surface and in transmission respectively (common scale bar). (C) shows the cell after the deposition of the Pt protective layer. (D) shows the cell after the cut while (E) shows an enlargement of the section of the cell with an enhanced contrast. (F) EDX spectra from the background (blue region in (E)) and from the bright spot marked with the red arrow in (E) are shown in blue and red respectively.
(TIF)

Figure S7 **Microscope pictures of figures S5 C to E where Ag signal was detected by EDX (feft column).** In the middle column, raw EDX data from AgNPs regions and background corresponding to each image on the left column. (B) Red line represents the EDX taken from the area indicated in (A), while the green line is the background from an arbitrary position in the cell. (D) represents the data from one of the areas with Ag signal shown in the image (C), while (E) is the background from the same cell. (G) EDX data from different Ag regions are represented with the black, green and red lines while the background is represented with the blue line. Right column: detection of bright spots interpreted as AgNPs without EDX signal. Examples of AgNPs are pointed out with bright blue arrows. Detection of AgNPs from figures S5 A and B are reported in details in Figs 4 and S6 respectively.
(TIF)

Table S1 Kernel of the Spatial Gaussian filter used to deconvolve the EDX signal from Ag and Os in Figure 4.
(DOCX)
