# Supplementary figures and images for: Detection of Silver Nanoparticles inside Marine Diatom Thalassiosira pseudonana by Electron Microscopy and Focused Ion Beam
Source: PLoS One. 2014 May 5;9(5):e96078. doi: 10.1371/journal.pone.0096078 (PMC4010438; doi:10.1371/journal.pone.0096078)

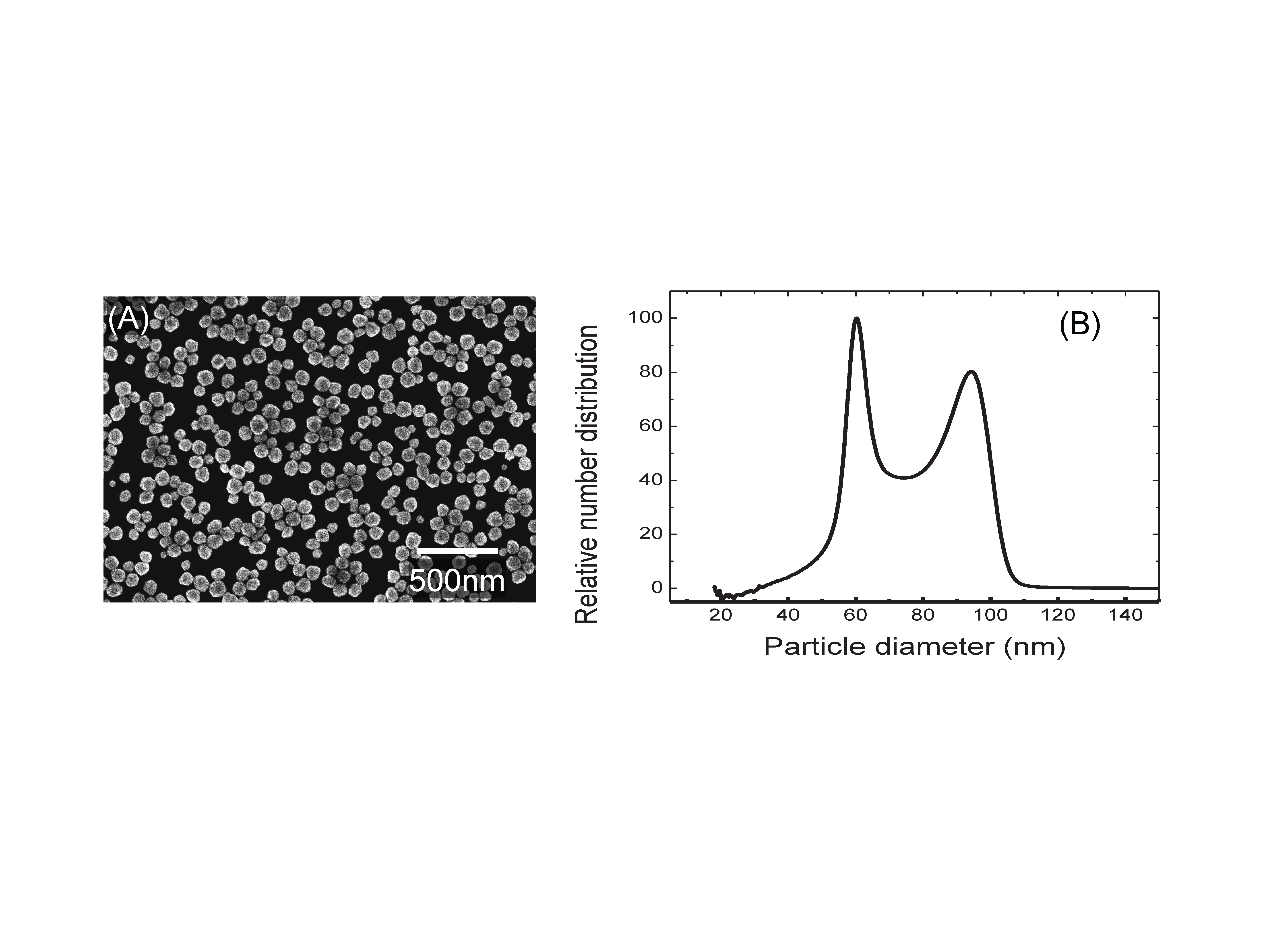

Supplement: Figure S1 — (A) SEM of the NPs used in the study. (B) Centrifuge Liquid Sedimentation analysis of the size of the AgNPs. Particles were analyzed using CLS and SEM. Particles' diameters resulted less than 120 nm. Below 30 nm CLS reaches its limit of sensitivity and we cannot exclude particles of smaller size. For this reason we considered our nanoparticles as polydispersed AgNPs. (TIF) [file pone.0096078.s001.tif]

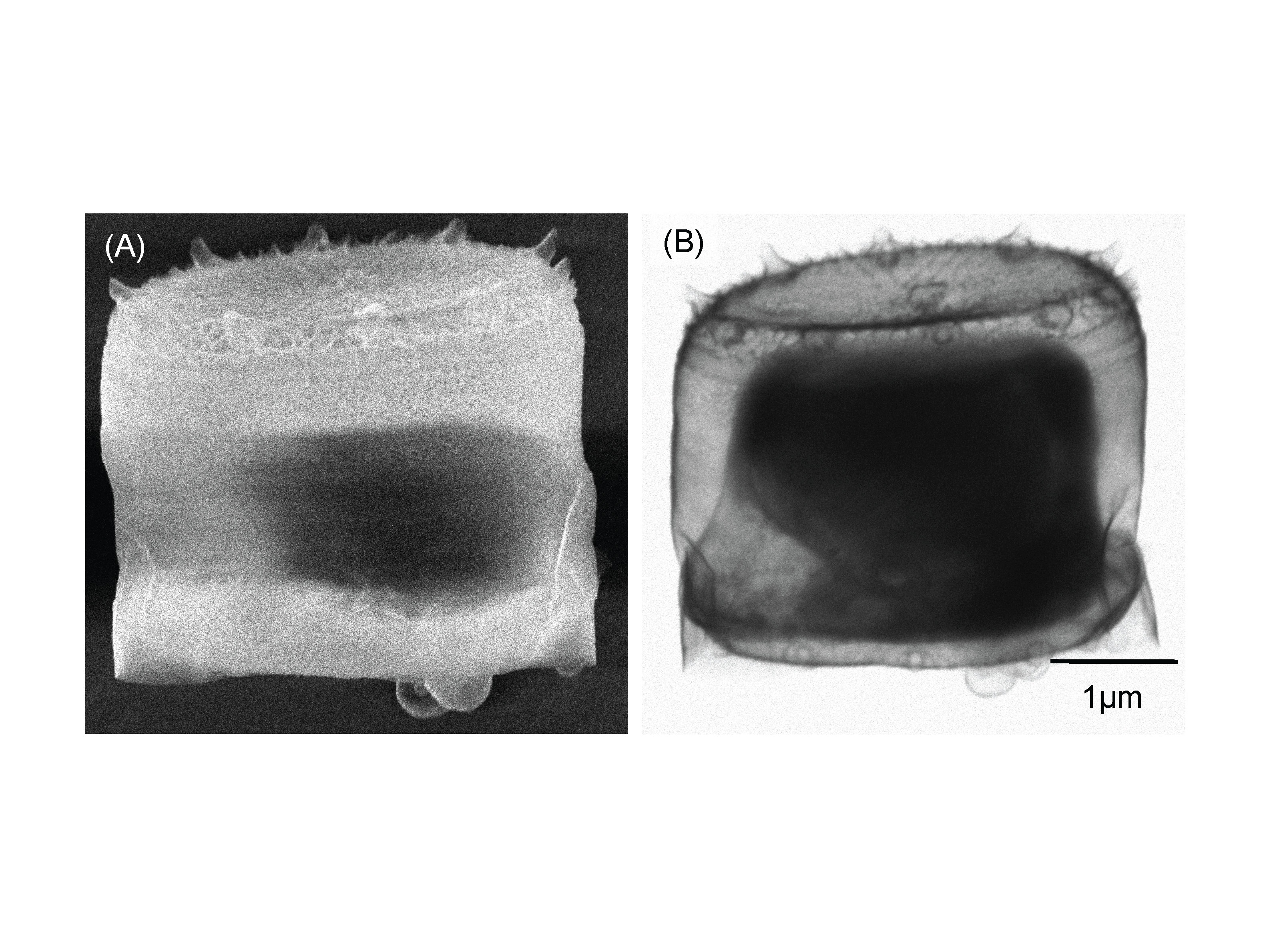

Supplement: Figure S2 — SEM images of one of the diatoms from the control sample using the signal from the surface (A) and in transmission (B) (common scale bar). The control sample was prepared in the same way described in materials and methods without the incubation with AgNPs. The shrinkage of the cells was calculated from STEM images in Figures 3B and S2B using the ratio of the volume of the shell and cell-membrane. The shape used to estimate both volumes was a cylinder (Lx2π(D/2)2), where L and D are the measured length and diameter of the diatom corresponding to the vertical and horizontal directions respectively in both pictures. (TIF) [file pone.0096078.s002.tif]

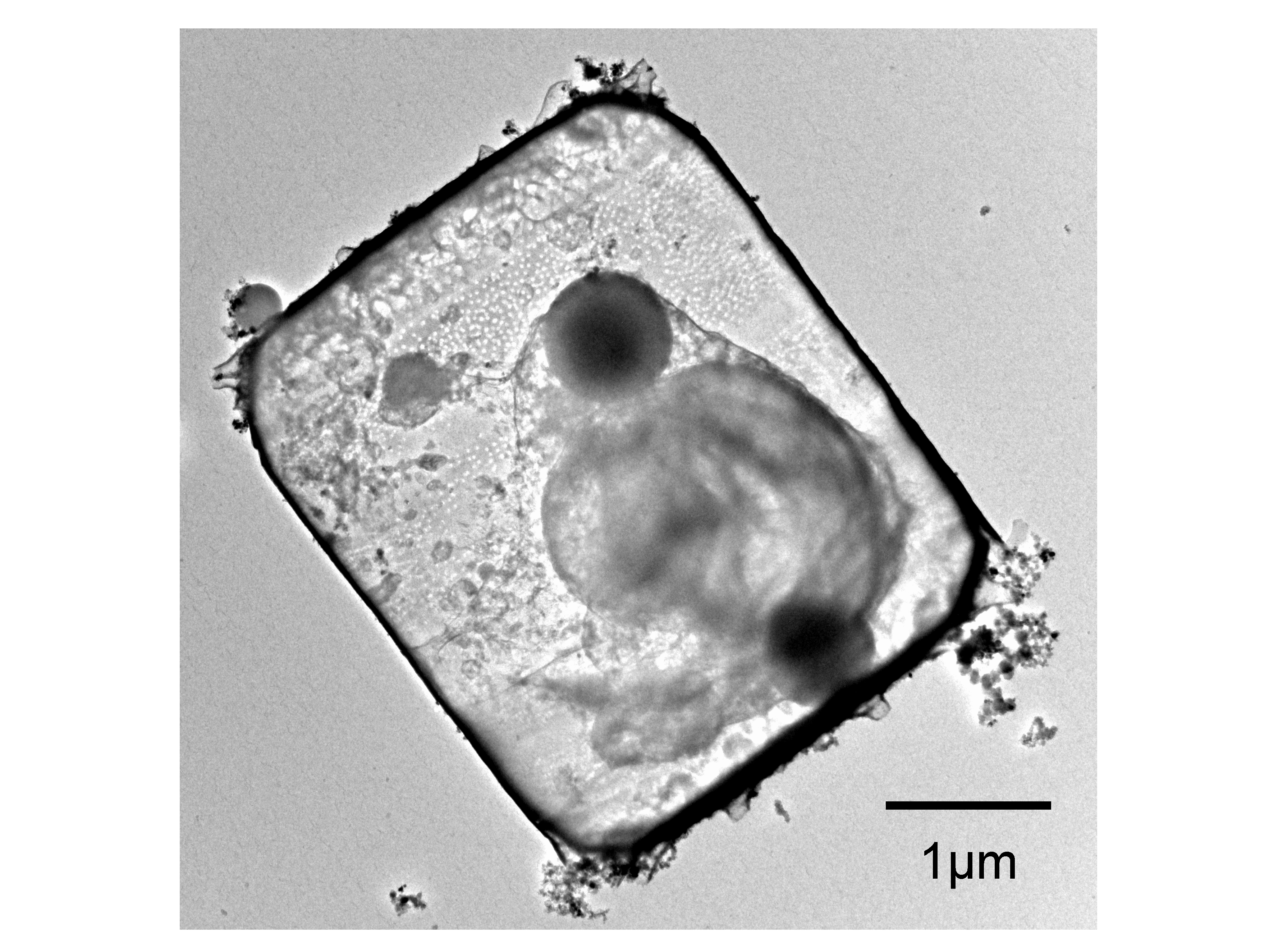

Supplement: Figure S3 — Transmission electron microscope (TEM) image performed with 200 keV HV. Picture S3 shows a TEM image of a AgNP exposed diatom. The picture was made using a JEOL JEM 2100 TEM microscope at 200 keV (see Figure S3). We compared the STEM images at 30 keV with the ones at 200 keV from the TEM. The contrast and resolution of TEM images were superior to STEM, but the main information about the localization of NPs was equivalent. The higher contrast in TEM images allows a better recognition of organelles in the diatom. (TIF) [file pone.0096078.s003.tif]

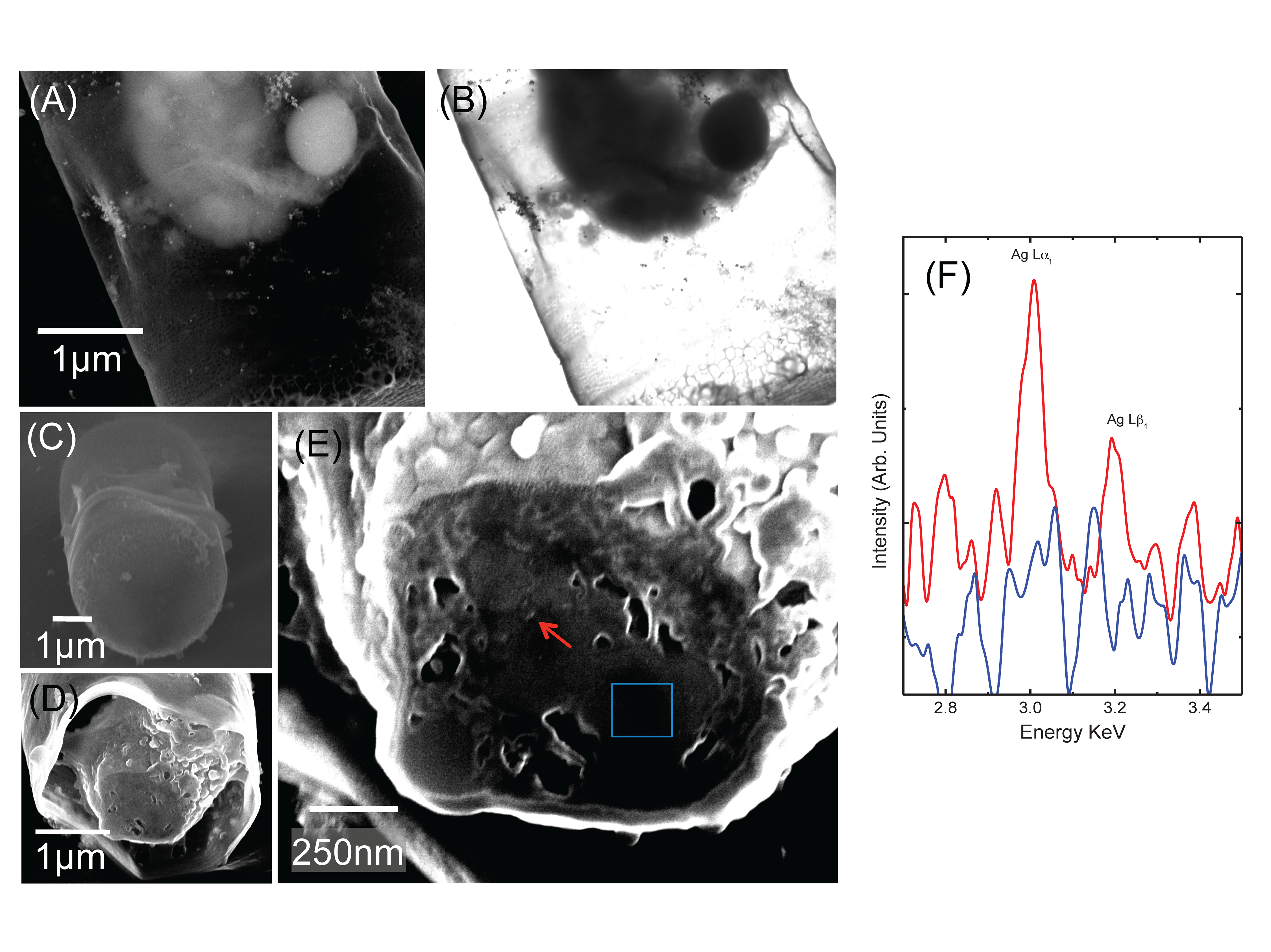

Supplement: Figure S4 — EDX Ag map before (A) and after (B) image processing using a Gaussian filter shown in the table S1. Images were processed using open software Image-J using the convolve command. (TIF) [file pone.0096078.s004.tif]

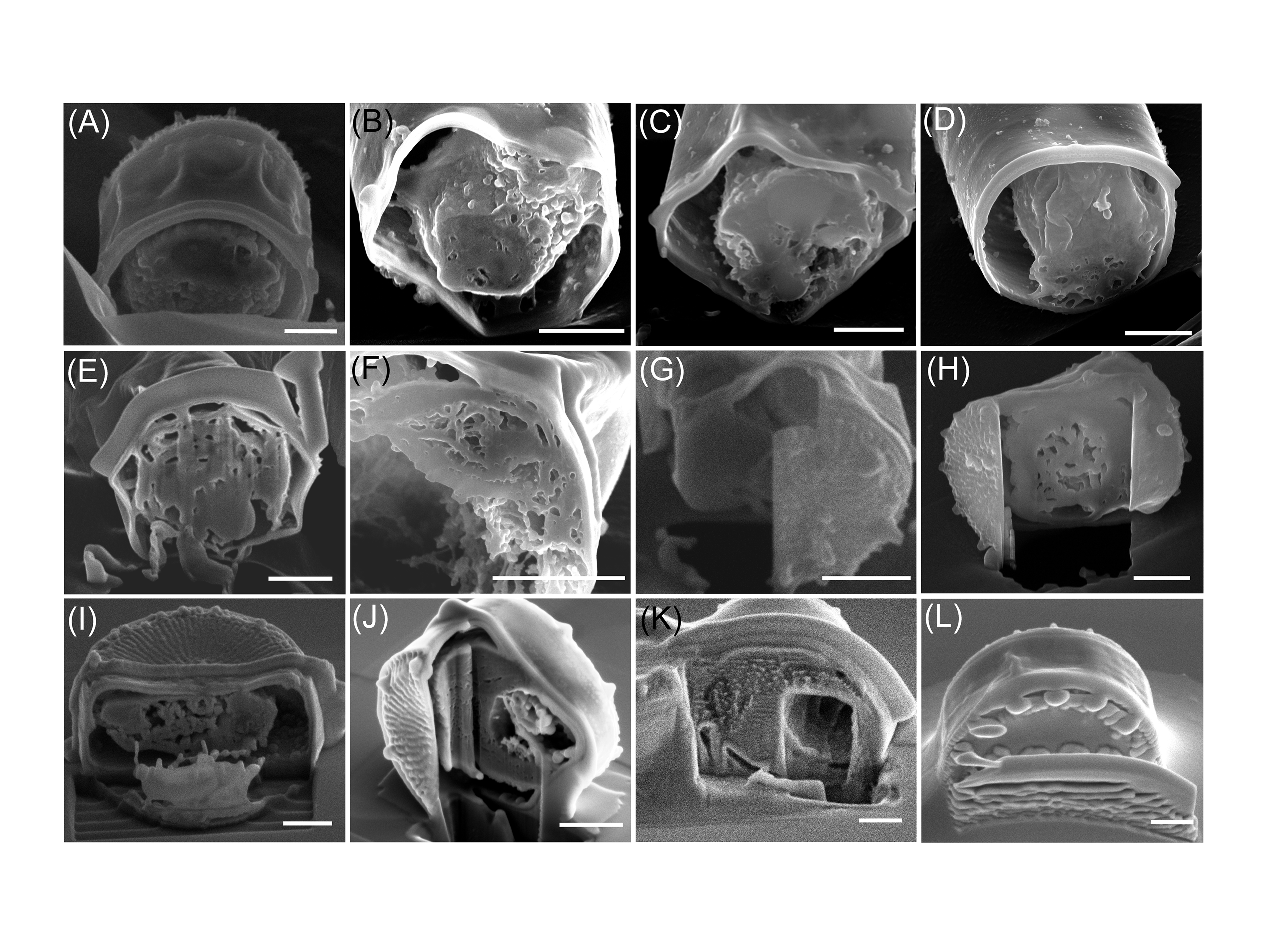

Supplement: Figure S5 — Electron microscope pictures of the total 12 cuts made in this study. Bright spots in the cytoplasm region of the section were found in a total of seven cases (A) to (G), while EDX signal associated with these spots were detected in five cases (A) to (E). We performed different cuts in a total of 12 diatom cells. Bright spots inside the cytoplasm of the cell, associated with high density clusters could be detected seven of the cuts, while Ag EDX signal associated with some of these spots was detected in five of them (a second detailed example is shown in Figure S6, while for the other cuts the raw data are summarized in S7). We attribute the fact that we see more bright spots than EDX signal to the higher cross section for electron-electron scattering than electron-X-rays scattering, and therefore there is a higher threshold for the detection of silver by EDX. In some cells no high density clusters or Ag EDX signal were visible inside the cell after the cut, even though the comparison of the intact cell by SEM/STEM suggested some of the NPs were not located at the surface. This could be partially explained by the introduction of damage by the ion abrasion, as evidenced by the detection of a curtain effect (the transport of material from the top of the cell). In these cases we did not detect neither silver nor the existence of high density spots. (TIF) [file pone.0096078.s005.tif]

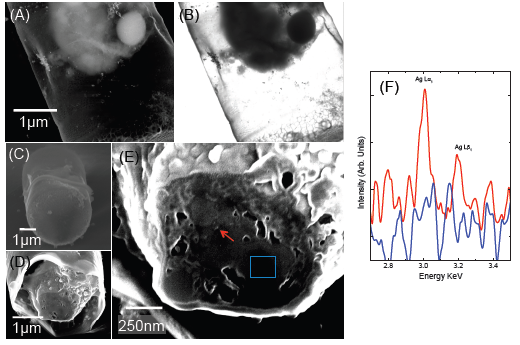

Supplement: Figure S6 — Sequence of the cut and detection of silver content into AgNPs exposed diatom. (A) and (B) are scanning electron microscope images of the diatom incubated with AgNPs using the signal from the surface and in transmission respectively (common scale bar). (C) shows the cell after the deposition of the Pt protective layer. (D) shows the cell after the cut while (E) shows an enlargement of the section of the cell with an enhanced contrast. (F) EDX spectra from the background (blue region in (E)) and from the bright spot marked with the red arrow in (E) are shown in blue and red respectively. (TIF) [file pone.0096078.s006.tif]

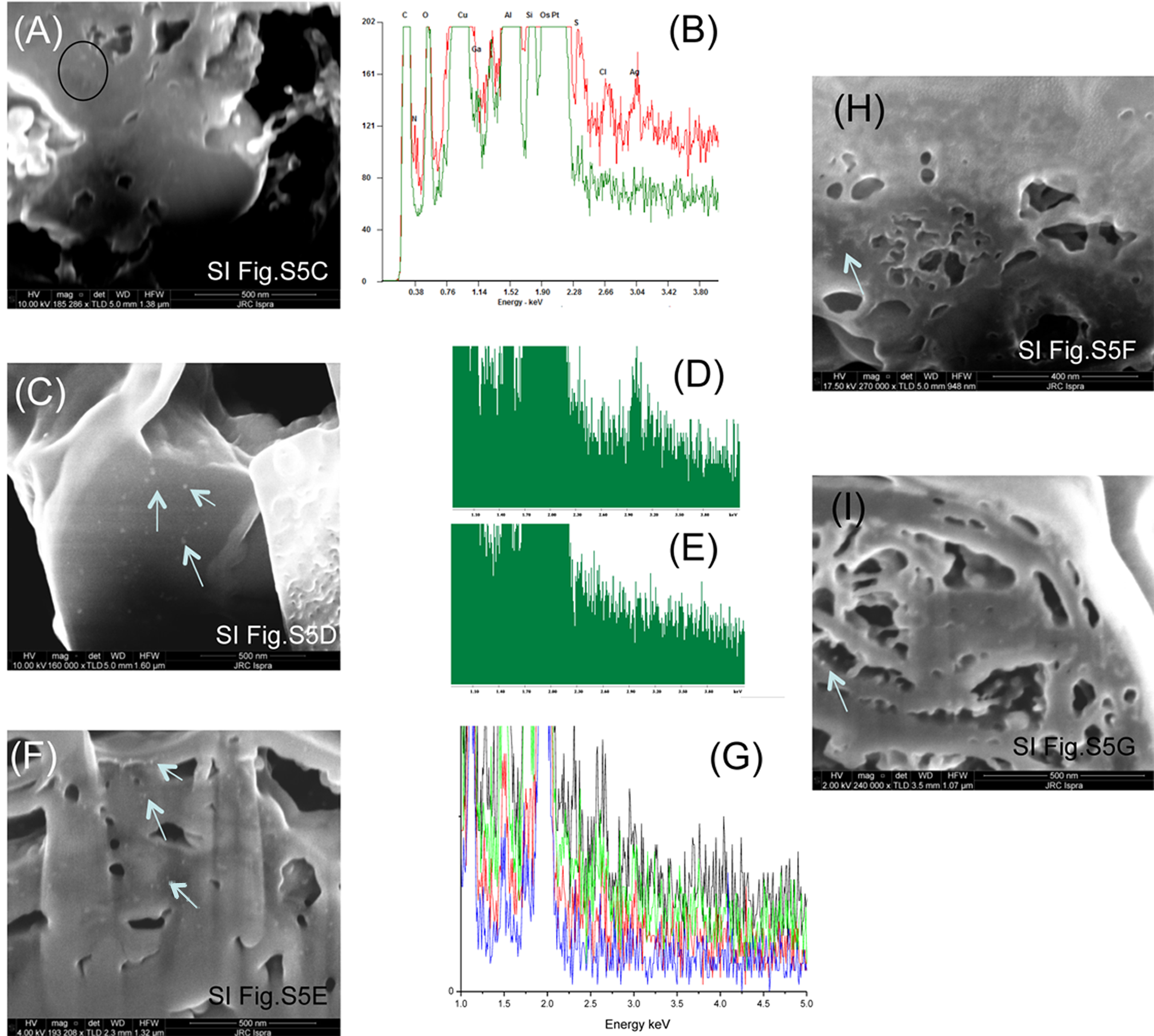

Supplement: Figure S7 — Microscope pictures of figures S5 C to E where Ag signal was detected by EDX (feft column). In the middle column, raw EDX data from AgNPs regions and background corresponding to each image on the left column. (B) Red line represents the EDX taken from the area indicated in (A), while the green line is the background from an arbitrary position in the cell. (D) represents the data from one of the areas with Ag signal shown in the image (C), while (E) is the background from the same cell. (G) EDX data from different Ag regions are represented with the black, green and red lines while the background is represented with the blue line. Right column: detection of bright spots interpreted as AgNPs without EDX signal. Examples of AgNPs are pointed out with bright blue arrows. Detection of AgNPs from figures S5 A and B are reported in details in Figs 4 and S6 respectively. (TIF) [file pone.0096078.s007.tif]
