# Supplementary material for: Detection of Silver Nanoparticles inside Marine Diatom Thalassiosira pseudonana by Electron Microscopy and Focused Ion Beam
Source: PLoS One. 2014 May 5;9(5):e96078. doi: 10.1371/journal.pone.0096078 (PMC4010438; doi:10.1371/journal.pone.0096078)
Supplement: Table S1 — Kernel of the Spatial Gaussian filter used to deconvolve the EDX signal from Ag and Os in Figure 4. (DOCX) [file pone.0096078.s008.docx]

| 0 | 1 | 2 | 1 | 0 |
| --- | --- | --- | --- | --- |
| 1 | 3 | 5 | 3 | 1 |
| 2 | 5 | 9 | 5 | 2 |
| 1 | 3 | 5 | 3 | 1 |
| 0 | 1 | 2 | 1 | 0 |

Table S1
